# Supplementary material for: Structural and Biochemical Insights into Bis(2-hydroxyethyl) Terephthalate Degrading Carboxylesterase Isolated from Psychrotrophic Bacterium Exiguobacterium antarcticum
Source: Int J Mol Sci. 2023 Jul 27;24(15):12022. doi: 10.3390/ijms241512022 (PMC10418727; doi:10.3390/ijms241512022)
Supplement: Supplementary file 1 [file ijms-24-12022-s001.zip › ijms-2526256-supplementary.pdf]

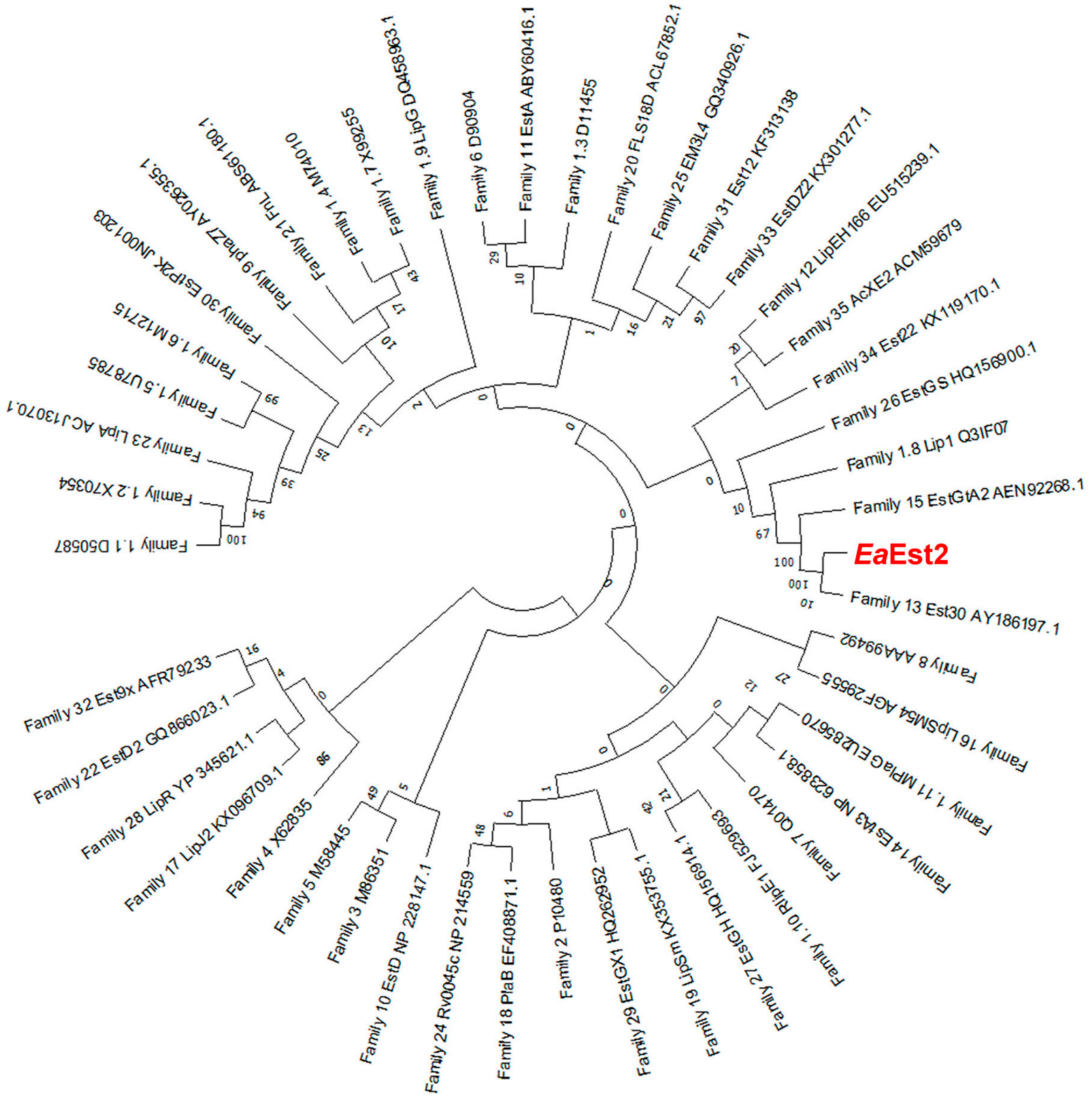

Figure S1. Phylogenetic tree of bacterial lipolytic enzymes. Protein sequences used for the classification of lipolytic enzymes based on the classification system [1] were aligned with Ea30 (*Ea*Est2) using an unrooted neighbor-joining tree by MEGA-X. A consensus tree following 500 bootstrap replications are shown. The percentage of replicate trees in which the associated taxa clustered together is shown at the branches' top. The family group, protein name, and the UniProt ID were shown.

Reference

[1] Hitch TCA, Clavel T: A proposed update for the classification and description of bacterial lipolytic enzymes. PeerJ 2019, 7:e7249.
